# Supplementary material for: Slippery when wet: cross-species transmission of divergent coronaviruses in bony and jawless fish and the evolutionary history of the Coronaviridae
Source: Virus Evol. 2021 May 31;7(2):veab050. doi: 10.1093/ve/veab050 (PMC8244743; doi:10.1093/ve/veab050)

**Supplementary Figure 1**. Agarose gel electrophoresis showing PCR products from two sets of primers (12P2 and 06P1; see Supplementary Table 1 for primer details) that targeted regions in coronavirus ORF1b (including the RdRp) for pouched lamprey. Samples “06”, “08”, and “12” here correspond to samples 1, 2 and 3 listed in Figure 2. Samples “exCT”, “07”, “NRT”, “CS29”, and “NEG” correspond to extraction-control, limitedly-affected-individual (kanakana letovirus not strongly detected), reverse-transcription-control, primer-specificity-control (DNA extract from *Geotria australis* individual), and the PCR-negative-control samples respectively. Sample 08 band is faint but present in the 06P1 PCR product.


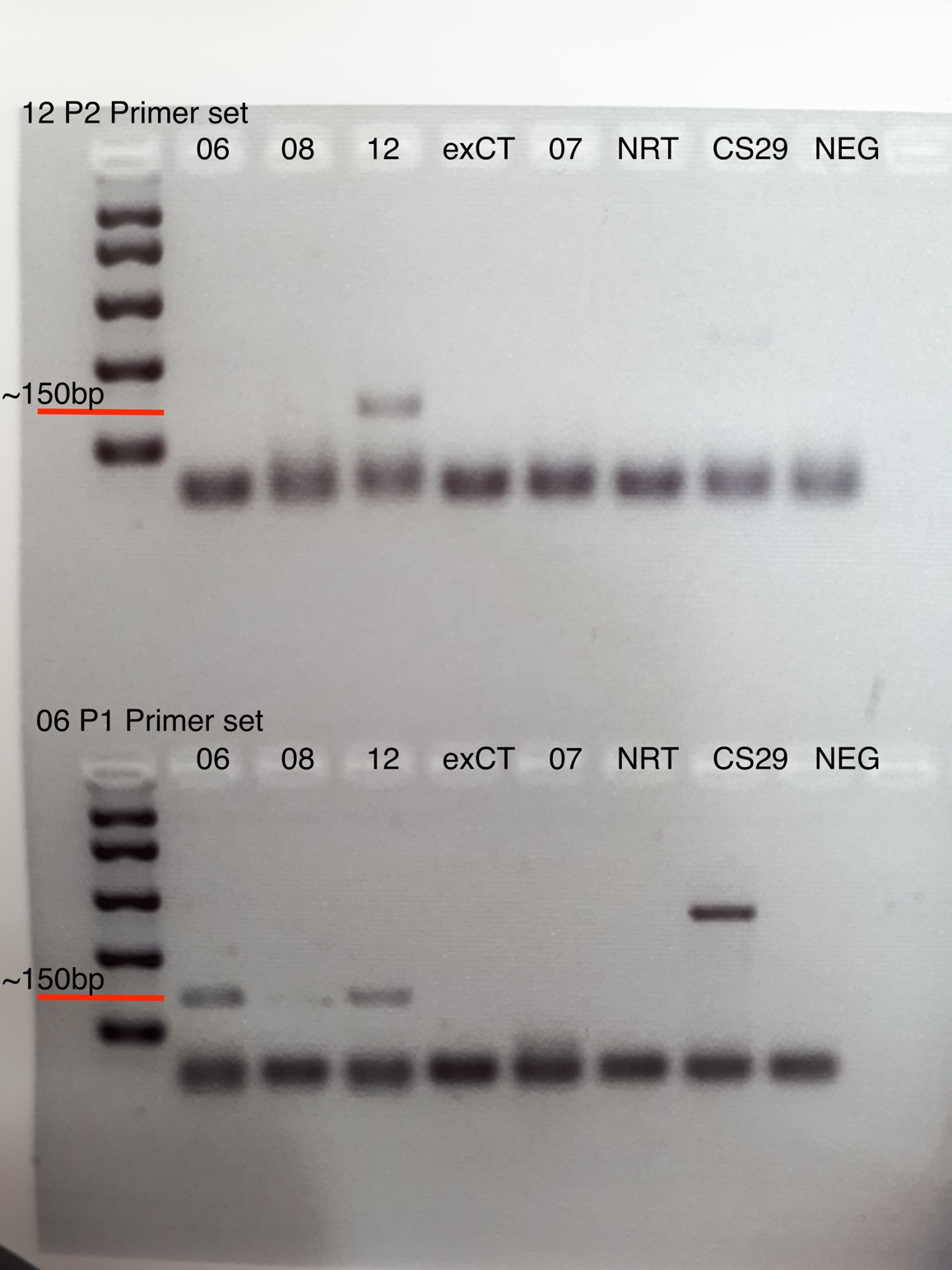

Supplement: veab050_Supp [file veab050_supp.zip › Supplementary Figure 1.docx]
