# Supplementary material for: Slippery when wet: cross-species transmission of divergent coronaviruses in bony and jawless fish and the evolutionary history of the Coronaviridae
Source: Virus Evol. 2021 May 31;7(2):veab050. doi: 10.1093/ve/veab050 (PMC8244743; doi:10.1093/ve/veab050)
Supplement: veab050_Supp [file veab050_supp.zip › Supplementary Table 1.docx]

**Supplementary Table 1**. List of primer sets used for the RT-PCR confirmation of kanakana letovirus in specimens of pouched lamprey.

| **Primer set** | **kanakana letovirus** | **Contig used** | **Transcript region (bp)** | **Product (bp)** | **5' Forward 3'** | **Tm (C)** | **5' Reverse 3'** | **Tm (C)** |
| --- | --- | --- | --- | --- | --- | --- | --- | --- |
| 06P1 | K. letovirus 1 | 06_TRINITY_DN122665_c0_g1_i1_len2572_ORF1b | 1179 to 1309 | 150 | TCATGGCCGTGAACAAACGA | 60.5 | CGAAGAAGGCTCGGCATTGA | 60.7 |
| 12P2 | K. letovirus 3 | 12_TRINITY_DN10196_c0_g1_i1_len5095_ORF1b | 3301 to 3451 | 150 | TCCTCGTTCCCAGTTGGAGT | 60.5 | CGCTGGAGTGTCCACATTCT | 60.0 |
