# Supplementary material for: Slippery when wet: cross-species transmission of divergent coronaviruses in bony and jawless fish and the evolutionary history of the Coronaviridae
Source: Virus Evol. 2021 May 31;7(2):veab050. doi: 10.1093/ve/veab050 (PMC8244743; doi:10.1093/ve/veab050)
Supplement: veab050_Supp [file veab050_supp.zip › Supplementary Table 2.docx]

**Supplementary Table 2**. GenBank accession numbers of viruses used in Figure 1.

| **Genomic region** | **NCBI Accession** | **Virus** |
| --- | --- | --- |
| ORF1ab | YP_003766 | Human alphacoronavirus NL63 |
|  | NP_828849 | SARS coronavirus Tor2 |
|  | YP_005352837 | White eye deltacoronavirus HKU16 |
|  | YP_001941164 | Turkey gammacoronavirus |
|  | AUE23860 | Atlantic salmon bafinivirus |
|  | YP_009130641 | Chinook salmon bafinivirus |
|  | NC_007447 | Bovine torovirus |
|  | YP_008798230 | Porcine torovirus |
|  | GECV01031551.1 | Microhyla letovirus |
|  | QEG08237 | Pacific salmon nidovirus |
| Spike | QBG64657 | Alphacoronavirus UKRn3 |
|  | 7CAB_C | SARS-CoV-2 |
|  | AIB07807 | Deltacoronavirus PDCoV/USA/Ohio137/2014 |
|  | ABW75138 | Turkey gammacoronavirus |
|  | AUE23862.1 | Atlantic salmon bafinivirus |
|  | YP_009130643.1 | Chinook salmon bafinivirus |
|  | QHN70902.1 | Bovine torovirus |
|  | QBJ02013.1 | Porcine torovirus |
|  | GECV01031551.1 | Microhyla letovirus |
|  | QEG08239.1 | Pacific salmon nidovirus |
