# Supplementary material for: Slippery when wet: cross-species transmission of divergent coronaviruses in bony and jawless fish and the evolutionary history of the Coronaviridae
Source: Virus Evol. 2021 May 31;7(2):veab050. doi: 10.1093/ve/veab050 (PMC8244743; doi:10.1093/ve/veab050)
Supplement: veab050_Supp [file veab050_supp.zip › Supplementary Table 3.docx]

**Supplementary Table 3**. Accession numbers of viruses used in Figure 3b.

| **Virus family** | **NCBI Accession** | **Virus** |
| --- | --- | --- |
| *Coronaviridae* | See Figure 3c | |
| *Tobaniviridae* | QBJ02070 | Porcine torovirus |
|  | YP_803213 | White bream virus |
|  | YP_009505581 | Fathead minnow nidovirus |
|  | YP_009130641 | Chinook salmon bafinivirus |
| *Arteriviridae* | YP_009118960 | African pouched rat arterivirus |
|  | AYF59232 | Equine arteritis virus |
|  | YP_009755852 | Wuhan japanese halfbeak arterivirus |
|  | YP_009755867.1 | Nanhai ghost shark arterivirus |
| *Roniviridae* | YP_001661452 | Gill associated virus |
|  | ACA21302 | Giant tiger prawn yellow head virus |
| *Mesoniviridae* | AGL73198 | Kamphang Phet virus |
|  | YP_004767305 | Nam Dinh virus |
| *Abyssoviridae* | YP_009553214 | Aplysia californica nido-like virus |
