# Supplementary figures and images for: Slippery when wet: cross-species transmission of divergent coronaviruses in bony and jawless fish and the evolutionary history of the Coronaviridae
Source: Virus Evol. 2021 May 31;7(2):veab050. doi: 10.1093/ve/veab050 (PMC8244743; doi:10.1093/ve/veab050)

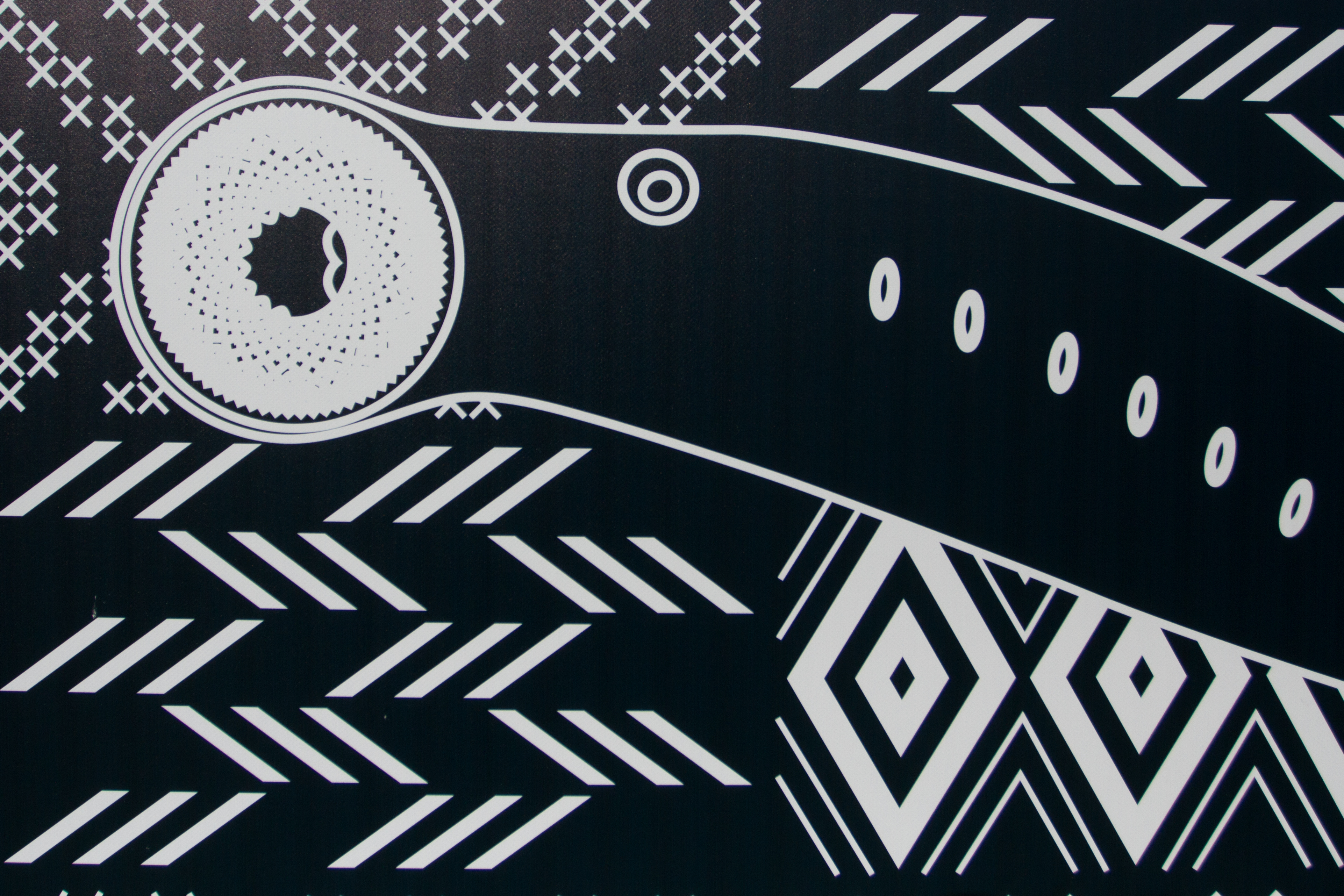

Supplement: veab050_Supp [file veab050_supp.zip › IMG_2916.jpg]

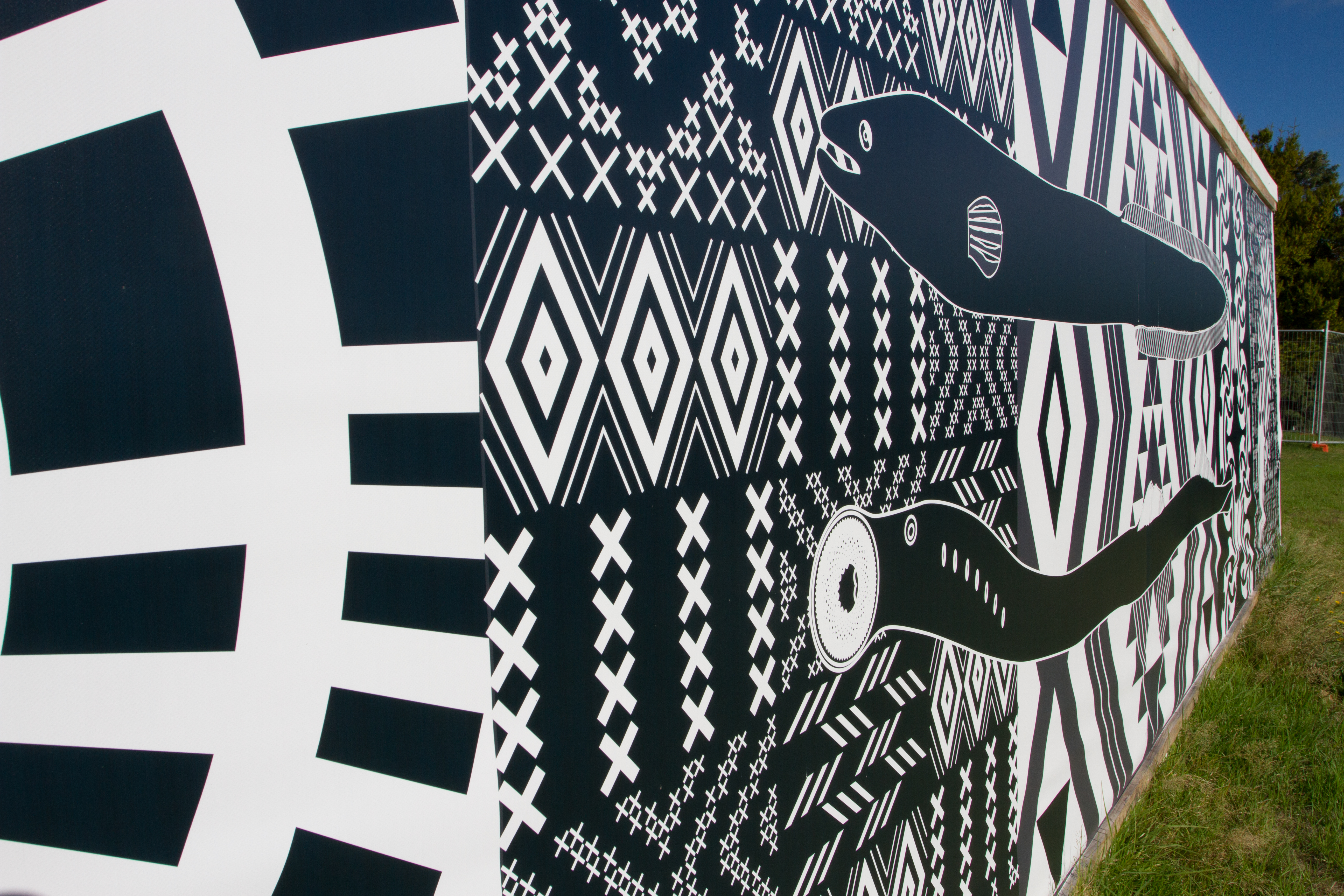

Supplement: veab050_Supp [file veab050_supp.zip › IMG_2918.jpg]

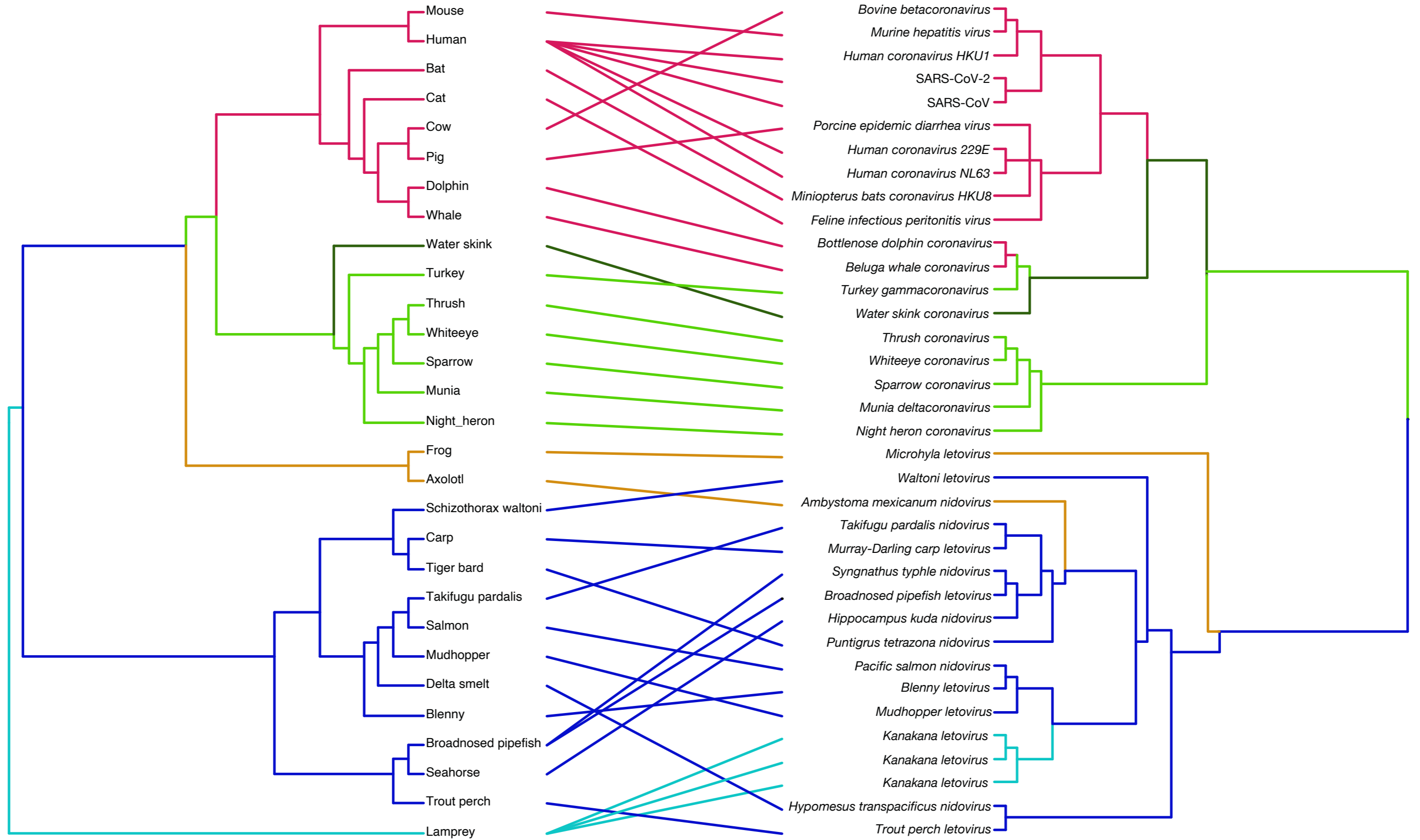

Supplement: veab050_Supp [file veab050_supp.zip › Supplementary Figure 2.pdf]
